# Dietary Intake of Free Sugars is Associated with Disease Activity and Dyslipidemia in Systemic Lupus Erythematosus Patients

**DOI:** 10.3390/nu12041094

**Published:** 2020-04-15

**Authors:** María Correa-Rodríguez, Gabriela Pocovi-Gerardino, José-Luis Callejas-Rubio, Raquel Ríos Fernández, María Martín-Amada, María-Gracia Cruz-Caparros, Irene Medina-Martínez, Norberto Ortego-Centeno, Blanca Rueda-Medina

**Affiliations:** 1 Department of Nursing, Health Sciences Faculty, University of Granada (UGR), Avenida de la Ilustración s/n, 18100 Granada, Spain; macoro@ugr.es (M.C.-R.); irenemedina@correo.ugr.es (I.M.-M.).; blarume@ugr.es (B.R.-M.); 2 Instituto de Investigación Biosanitaria, IBS, Avda, de Madrid, 15, Pabellón de consultas externas 2, 2ª planta, 18012 Granada, Spain; nortego@gmail.com; 3 Unidad de Enfermedades Autoinmunes Sistémicas, Servicio de Medicina Interna, Hospital Universitario San Cecilio, Av, de la Investigación, s/n, 18016 Granada, Spain; jlcalleja@telefonica.net (J.-L.C.-R.); rriosfer@hotmail.com (R.R.F.); 4 Unidad de Enfermedades Autoinmunes Sistémicas, Servicio de Medicina Interna, Complejo Hospitalario de Jaén, Av. del Ejército Español, 10, 23007 Jaén, Spain; madfmar@gmail.com; 5 Unidad de Enfermedades Autoinmunes Sistémicas, Servicio de Medicina Interna, Hospital de Poniente, Carretera de Almerimar, 31, 04700 El Ejido, Almería, Spain; grcrca@hotmail.com

**Keywords:** autoimmune disease, lupus, sugar intake, free sugars intake, inflammation, cardiovascular factors

## Abstract

Diet has been closely associated with inflammatory autoimmune diseases, including systemic lupus erythematosus (SLE). Importantly, the consumption of dietary sugars has been positively linked to elevated levels of some inflammation markers, but the potential role of their consumption on the prognosis of autoimmune diseases has not yet been examined. The aim of this study was to evaluate the association between the dietary intake of free sugars and clinical parameters and cardiovascular (CVD) risk markers in patients with SLE. A cross-sectional study including a total of 193 patients with SLE (aged 48.25 ± 12.54 years) was conducted. The *SLE Disease Activity Index* (SLEDAI-2K) and the *SDI Damage Index* were used to asses disease activity and disease-related damage, respectively. Levels of C-reactive protein (CRP; mg/dL), homocysteine (Hcy; µmol/L), anti-double stranded DNA antibodies (anti-dsDNA) (IU/mL), complement C3 (mg/dL), and complement C4 (mg/dL), among other biochemical markers, were measured. The main factors we considered as risk factors for CVD were obesity, diabetes mellitus, hypertension, and blood lipids. The dietary-intrinsic sugar and added-sugar content participants consumed were obtained via a 24-h patient diary. Significant differences were observed in dietary sugar intake between patients with active and inactive SLE (in grams: 28.31 ± 24.43 vs. 38.71 ± 28.87; *p* = 0.035) and free sugar intake (as a percentage: 6.36 ± 4.82 vs. 8.60 ± 5.51; *p* = 0.020). Linear regression analysis revealed a significant association between free sugars intake (by gram or percentage) and the number of complications (β (95% CI) = 0.009 (0.001, 0.0018), *p* = 0.033)); (β (95% CI) = 0.046 (0.008, 0.084), *p* = 0.018)), and SLEDAI (β (95% CI) = 0.017 (0.001, 0.034), *p* = 0.043)); (β (95% CI) = 0.086 (0.011, 0.161), *p* = 0.024)) after adjusting for covariates. Free sugars (g and %) were also associated with the presence of dyslipidaemia (β (95% CI) = −0.003 (−0.005, 0.000), *p* = 0.024)) and (β (95% CI) = −0.015 (−0.028, −0.002), *p* = 0.021)). Our findings suggest that a higher consumption of free sugars might negatively impact the activity and complications of SLE. However, future longitudinal research on SLE patients, including dietary intervention trials, are necessary to corroborate these preliminary data.

## 1. Introduction

Systemic autoimmune diseases are a broad range of related diseases characterised by immune system dysregulation which results in inappropriate inflammation and damage to multiple tissues. Among autoimmune diseases in general, systemic lupus erythematosus (SLE) is characterised by a loss of tolerance to self-antigens and the production of high titres of serum autoantibodies [1]. Inflammation processes in SLE result in the increase in inflammatory mediators that promote accelerated atherosclerosis, endothelium damage, and SLE progression [2].

The pathogenesis of inflammatory dysregulation underlying autoimmune diseases remains unknown [3], although it has been proposed that genetic, epigenetic, and environmental factors are determinants in the development and prognosis of these diseases [4]. Diet has been closely associated with inflammatory autoimmune diseases and is among the modifiable factors related to them [5]. In fact, previous studies have established nutrients as influential factors in inflammatory autoimmune diseases such as SLE or rheumatoid arthritis [6,7]. However, no conclusive associations have yet been consistently established and therefore, the links between diet and SLE remain elusive and intriguing.

Dietary sugar, which is consumed in high amounts in the standard Western diet, contributes to increased subclinical inflammation [8,9,10,11]. O’Connor et al. reported that higher intakes of free sugar was positively linked to some inflammation markers in a population-based study of adults [12]. Similarly, the excessive consumption of free sugars has been implicated in the increased risk of various diseases characterised by the presence of chronic low-grade inflammation, including obesity, diabetes, and metabolic syndrome [13]. Nevertheless, the potential role of the consumption of free sugar in autoimmune diseases has not yet been examined.

Considering that sugar consumption can modulate inflammation [11], and taking into account that patients with SLE have an increased cardiovascular risk because this disease results in accelerated atherosclerotic processes [14], new research investigating the relationship between free sugars and SLE is especially relevant. Thus, we aimed to investigate the possible association of free sugars dietary intake and SLE activity, damage accrual, and cardiovascular disease (CVD) risk markers in a population of lupus patients.

## 2. Materials and Methods

### 2.1. Study Population

A cross-sectional study was conducted among a population of SLE patients recruited in the Andalusian region of Spain at three public outpatient clinics of systemic autoimmune diseases. All patients met the SLE revised criteria of the American College of Rheumatology (ACR) or Systemic Lupus Erythematosus International Collaborating Clinics Group (SLICC) criteria [15,16]. The participants were diagnosed for SLE at least one year prior to the study, were clinically stable without changes in the Systemic Lupus Erythematosus Disease Activity Index 2000 (SLEDAI-2K) [17] and/or in drug therapy over the six months just prior to the study. The exclusion criteria were: pregnancy, cerebrovascular disease, ischemic heart disease, active infections, major trauma or surgery in the previous six months, serum creatinine ≥ 1.5 mg/dl and the presence of other autoimmune and/or chronic diseases not related with the main disease (i.e., type 1 diabetes, multiple sclerosis rheumatoid arthritis, cancer).

A total of 193 SLE patients met the inclusion criteria and were included in the study after giving written informed consent. The study participants had a mean age of 48.25 ± 12.54 and most were female (90.2%). Local ethics committees approved the study protocol (“Comité Coordinador de Ética de la Investigación en Andalucía” (30-11-2016)) that was conducted in agreement with the Declaration of Helsinki.

In an in-person medical consultation, data about participant´s clinical history comprising actual medical treatment were obtained. Next, food and beverage intake and body mass index (BMI) were assessed through an in-person consultation with a nutritionist-dietitian.

### 2.2. SLE Disease Activity Index (SLEDAI) and Damage Index for Systemic Lupus Erythematosus (SDI) Assessment

The activity of the disease was assessed with the Systemic Lupus Erythematosus Disease Activity Index 2000 (SLEDAI-2K) [17]. SLE Disease Activity Index (SLEDAI) includes 24 items, 8 of which are laboratory results and 16 are clinical items. The items are scored on the basis of the presence or absence of these manifestations in the previous 10 days. The total score of results is calculated from the sum of all 24 descriptors (scoring range 0 to 105; clinical relevance: score of 6). SLEDAI-2K is a modification of SLEDAI that permits the evaluation of ongoing SLE activity in some clinical items as proteinuria, alopecia, mucosal ulcers and skin rash, instead of only new occurrence as defined in the original SLEDAI [17]. A reduction in SLEDAI-2 K of 4 is defined as a meaningful improvement [18].

The SLICC/ACR (SDI) damage index was used to measure disease-related organ damage [19]. With this instrument the irreversible damage is assessed in SLE patients, independently of its cause (maximum scoring: 47) [20]. The SDI score progressively increases over time and patients showing higher SDI scores early in the progression of disease have been related with a worse prognosis and augmented mortality [21].

### 2.3. Definition of Remission

Based on the DORIS framework [22], clinical Remission was defined at each visit as a SLEDAI < 2, absence of anti-DNA antibodies, corticoids use ≤5 mg/day and maintenance treatment with antimalarials.

### 2.4. Assay of Anti-Double Stranded DNA Antibodies (Anti-dsDNA) and Complement Levels

A BioPlex 2200 System (Bio-Rad, Hercules, CA, USA) which automatically detects antibodies to several antigens in one tube, was used to measure Anti-dsDNA titres. The cut-off values expressed in IU/mL and established according to manufacturer instructions were: 5–9 IU/mL (indeterminate) and ≥ 10 IU/mL (positive). Serum samples were obtained to determine quantitatively human complement components C3 and C4 by immunoturbidimetric assay (Beckman Coulter AU System C-reactive protein (CRP) Latex reagent) using a Beckman Coulter analyser (AU5800 Analyzer, Beckman Coulter, CA, USA). Ranges of 90–180 mg/dL and 10–40 mg/dL were considered as normal for C3 and C4, respectively.

### 2.5. High-Sensitivity C-Reactive Protein (hs-CRP) and Homocysteine (Hcy) Determinations

A highly sensitive technique based on immunoturbidimetric assays was used to measure hs-CRP levels (Beckman Coulter AU System CRP Latex reagent) in a Beckman Coulter analyser (AU5800 Analyzer, Beckman Coulter, CA, USA). Following the recommendations of the American Heart Association guidelines for assessment of cardiovascular risk, two categories for hs-CRP levels were considered: low to normal/average risk (cut-off point of 3: ≤ 3.0 mg/L) and high risk of cardiovascular disease (>3.0 mg/L) [23].

An enzymatic colorimetric assay was used to determine Hcy serum levels in a Beckman Coulter analyser (AU680, Beckman Coulter, CA, USA) with the Axis-Shield Liquid Stable (LS) 2-Part Homocysteine Reagent (Axis-Shield Diagnostics Ltd., Dundee, UK). The method used measures Hcy levels ranging between 2 and 44 µmol/L. A range between 5 and 15 µmol/L is considered as the normal laboratory reference levels, whereas for patients with an increased risk of CVD, such as SLE patients, values of < 10 µmol/L are recommended [24].

### 2.6. Cardiovascular Risk Factors

#### 2.6.1. Comorbidities

The occurrence of dyslipidaemia, obesity, hypertension, and/or type II diabetes, were considered cardiovascular risk factors. The presence of type II diabetes, either among patients with a previous diagnosis requiring drug therapy or diagnosed by clinicians (defined as a fasting blood glucose level of ≥126 mg/dL), was recorded. Patients were regarded as being hypertensive when their blood pressure was ≥140/90 mmHg, both for a previous clinical diagnosis or when antihypertensive therapy had been initiated. Body mass index was used to define obesity as a BMI of 25 kg/m^2^ or more. Participants were considered dyslipidaemic when at least two of their blood lipid parameters exceeded the following ranges: triglycerides [TGs] ≥ 150 mg/dL, low-density lipoprotein cholesterol [LDL-C] ≥ 140 mg/dL and high-density lipoprotein cholesterol [HDL-C] < 40 mg/dL) or if the patient had a previous diagnosis of dyslipidaemia and was receiving treatment with statins.

#### 2.6.2. Blood Lipid Profile

After an overnight fast, venous blood samples were drawn between 7:30 a.m. and 10:00 a.m. Afterwards, to obtain serum, samples were centrifuged for 15 min. Serum samples were immediately analysed using conventional laboratory methods to determine the total cholesterol (TC), LDL-C, HDL-C, and TG biochemical variables used here as cardiovascular risk factors.

#### 2.6.3. Ankle–Brachial Index (ABI) and Blood Pressure

The ABI, recognised as a predictive factor for cardiovascular events and all-cause mortality, was determined according to the American Heart Association recommendations to evaluate the presence of subclinical atherosclerosis. The same investigator measured systolic blood pressures (SBPs) on both arms and legs in the brachial arteries and at the ankle level, with a manual sphygmomanometer (Riester 1312 minimus^®^ II. Jungingen, Germany) and a portable vascular Doppler (Hadeco Minidop ES-100VX. Kawasaki, Japan). To calculate the ABI in each leg, a division of the highest ankle artery pressure by the highest brachial arterial systolic pressure of both arms was performed. The lowest ABI value was considered for the study. Blood pressure was measured using a Dinamap vital signs monitor (model BP 8800 Critikon, Inc., Tampa, FL, USA), following the European Heart Society recommendations.

### 2.7. Dietary Intake Assessment

After medical consultation, a qualified nutritionist/dietitian conducted an in-person interview. To estimate dietary intake including carbohydrates, starch and total sugars a 24-h diet recall was used. Data about servings and quantities of each food or beverage item consumed, recipe features (amount and number of ingredients), method of meal preparation, as well as intakes of sweets, alcoholic and non-alcoholic drinks, and added sugar in the last 24 h were recorded for each patient. Pictorial food models and standard household measures were used for a better exactness of the food quantities registered [25]. To convert the reported food and beverage intake into nutrient intake the software “El Alimentador” (Fundación Alimentación Saludable, Madrid, Spain) [26] was used. It should be noted that this program is based on the European and American Food composition tables and databases (FCTs/FCDBs) Additionally, it is also based on other sources such as food labelling and references provided by Spanish manufactures [27]. We obtained also information about total energy intake and alcohol intake from this 24-h recall.

### 2.8. Sugar Food Quantification and Classification

The following foods and beverages were considered as no having free sugars (“intrinsic” sugars): (1) fresh and unprocessed foods without labelling and any added ingredient: most meats, fresh fruits, fish, vegetables, etc.; (2) packaged/labeled foods in which the addition of free sugars was not included in the list of ingredients. In contrast, foods and beverages with added sugars comprised (1) all those packaged/labelled foods including in their ingredients list some form of “free sugars”. Then, the natural/intrinsic sugars and added sugars content were calculated.

### 2.9. Statistical Analysis

All statistical analyses were conducted using the SPSS^®^ Statistics version 21.0 (SPSS, Chicago, IL, USA) software. Categorical variables were expressed as frequencies and percentages and continuous variables as mean ± standard deviation. To verify data distribution normality, the Kolmogorov–Smirnov test was applied. Data were classified in three groups according to SLEDAI (active SLE: SLEDAI ≥ 5 and inactive SLE: SLEDAI < 5) and DORIS framework (clinical remission). To compare the study groups, Student’s t-test for continuous data and χ^2^ test for categorical data were used. Multivariate regression analysis was used to analyse the association between clinical disease variables and cardiovascular risk factors and free sugars consumption after adjusting for the following covariates: age, sex, energy intake and medical treatments (immunosuppressants, antimalarials and/or corticosteroids). Multivariate regression analysis was also performed separately by gender after adjusting for age, energy intake and medical treatment. P values of <0.05 were taken as statistically significant.

## 3. Results

Table 1 shows the main characteristics of the study population, both for the complete sample and stratified by active, inactive SLE and clinical remission at the time of the study. A total of 32 patients (16.58%) were classified as active SLE when the study was carried out, leaving 161 (83.41%) with inactive SLE. Of note, 74% of the patients were taking antimalarials, 33.9% immunosuppressants, and 39.1% corticosteroids. Moreover, significant differences in hs-CRP levels were detected between active and inactive SLE patients (*p* < 0.001). In addition, significant differences in the number of complications and anti-dsDNA levels were found between patients with active SLE and patients on clinical remission (*p* = 0.027 and *p* = 0.002, respectively). Regarding nutritional data, we found significant differences among patients having active and inactive SLE in terms of the intake of total free sugars (in grams: 28.31 ± 24.43 vs. 38.71 ± 28.87; *p* = 0.035) and free sugars (as a percentage: 6.36 ± 4.82 vs. 8.60 ± 5.51; *p* = 0.020). Furthermore, there were differences between patients with active SLE and clinical remission regarding total carbohydrates (196.84± 56.81 vs. 159.66 ± 45.82; *p* = 0.009) and total sugars (85.34 ± 31.26 vs. 68.70 ± 27.93; *p* = 0.042).

Descriptive characteristics of patients with active and inactive SLE and clinical remission according to gender are presented in Table 2. Note that significant differences between females with active and inactive SLE in terms of the intake of total free sugars (in grams: 40.77 ± 29.45 vs. 26.89 ± 24.26; *p* = 0.007) and free sugars (as a percentage: 9.09 ± 5.54 vs. 6.24 ± 4.88; *p* = 0.006) were observed.

Multivariate regression analysis showed that free sugar (in g) was significantly associated with the number of complications (β (95% CI) = 0.009 (0.001, 0.0018), *p* = 0.033)) and SLEDAI score (β (95% CI) = 0.017 (0.001, 0.034), *p* = 0.043)) in patients with SLE after adjusting for age, sex, energy intake, and medical treatment (Table 3). Similarly, free sugar (%) was also significantly associated with the number of complications (β (95% CI) = 0.046 (0.008, 0.084), *p* = 0.018)) and SLEDAI score (β (95% CI) = 0.086 (0.011, 0.161), *p* = 0.024)) in patients with SLE, after adjusting for covariates. In females, free sugar (in g) was significantly associated with the number of complications (β (95% CI) = 0.010 (0.000, 0.0019), *p* = 0.039)) and SLEDAI score (β (95% CI) = 0.023 (0.005, 0.041), *p* = 0.0012)) after adjusting for age, sex, energy intake, and medical treatment. Free sugar (%) was also significantly associated with the number of complications (β (95% CI) = 0.048 (0.007, 0.089), *p* = 0.021)), SLEDAI score (β (95% CI) = 0.109 (0.032, 0.187), *p* = 0.006)) and levels of complement C4 (β (95% CI) = −0.342 (−0.675, −0.010, p = 0.044)) in females with SLE after adjusting for covariates.

Regarding the cardiovascular risk factors, multivariate regression analysis revealed that free sugar consumption (both as an absolute amount in grams or as a percentage) was significantly associated with the presence of dyslipidaemia (β (95% CI) = −0.003 (−0.005, 0.000), p = 0.024)); (β (95% CI) = −0.015 (−0.028, −0.002), p = 0.021)) (Table 4). Note that free sugar (in g) and free sugar (in %) were significantly associated with the presence of dyslipidaemia (β (95% CI) = −0.004 (−0.007, 0.000), p = 0.026)); (β (95% CI) = −0.015 (−0.028, −0.001), p = 0.033)) in females.

## 4. Discussion

In this study we evidenced that the consumption of free sugars is higher in SLE patients with active disease compared to those with inactive SLE. Furthermore, we found that patients with active SLE have higher intakes of total carbohydrates and total sugars than patients on clinical remission. Additionally, we found a significant association between the dietary intake of free sugars and disease activity (measured using the SLEDAI) as well as between the number of complications and the presence of dyslipidaemia in SLE patients. This supports the hypothesis that the consumption of free sugar might exert a deleterious effect on the disease activity of SLE.

Available evidence suggests that dietary intake of sugars, especially free sugars, may stimulate subclinical inflammation [8,9,10]. For example, a prospective study including a large cohort of men concluded that the consumption of sugar-sweetened beverages was associated with an increased risk of coronary heart disease and some adverse changes in inflammatory factors [8]. Similarly, using data from the National Health and Nutrition Examination Survey, Hert et al. reported that the consumption of sugar-sweetened beverages was associated with the presence of biomarkers for chronic disease risk, independently of demographic and lifestyle factors [10]. Additionally, the consumption of added sugars has been associated with an increased risk of dyslipidaemia [13,28,29] and Welsh et al. reported a statistically significant correlation between dietary added sugars and dyslipidaemia among US adults [28]. Interestingly, the lack of association between dietary proteins and fats support the significance of free sugars compared to other nutrients in the disease activity of SLE patients. The evidence from our study is in line with these previous studies and suggests that the intake of free sugar may play a role in the inflammatory process underlying autoimmune disease.

To the best of our knowledge, this is the first study to examine the association between clinical disease activity variables and the dietary intake of free sugars in an autoimmune condition. The relationship between dietary sugar intake and autoimmunity has not been previously examined but prior studies have indicated that dietary factors play a key role in various autoimmune inflammatory diseases such as rheumatoid arthritis or inflammatory bowel disease [6,30,31,32]. Moreover, in agreement with our results, recently published research concluded that, in patients with SLE, a good diet can contribute to increasing the period of remission, preventing the adverse effects of medications, and improving the physical and mental well-being of patients [7]. Thus, dietary therapy has now been recognised as a promising approach to treating SLE, owing to both its potential prophylactic effects and its contribution to reducing co-morbidities and improving quality of life in these individuals [33].

The modulation of inflammatory processes may be an important factor in patients with SLE that could explain the deleterious effect of the consumption of high levels of free sugar. It is thought that the resulting reduction in disease activity may be secondary to the mechanisms that decrease inflammation and activate autoimmunity pathways [34,35]. Furthermore, it has been reported that dietary sugar may promote de novo synthesis of free fatty acids (FFA) in the liver [36,37], which may produce FFA metabolites, thus triggering inflammatory processes and the formation of reactive oxygen species [38]. In addition, it is possible that T helper cells are central players in mechanisms linking dietary factors to the modulation of autoimmune pathologies [5]. There is also growing evidence to suggest that dietary changes can alter the immune system and influence the onset and/or severity of autoimmune diseases such as SLE by affecting both the composition and function of gut microbial communities [39]. Indeed, Do et al. recently found that the dietary intake of high levels of glucose or fructose modulate the gut microbiota increasing intestinal permeability, which leads to inflammation, the development of metabolic endotoxemia, and lipid accumulation [40].

Finally, this study had some limitations that should be addressed. Firstly, it was cross-sectional study and, as such, was subject to the limitations inherent to this type of design. Secondly, there were also certain limitations derived from the use of the 24-h diet recall technique because it is prone to under-reporting and relies on participant memory [41]. Nevertheless, it should be noted that to minimise recall bias, face-to-face interviews were conducted by a well-trained researcher and the patients were also educated in this technique before their participation in the study. Additionally, although a 24-h recall is a valid assessment tool for estimating the intake of key nutrients including added sugars [42], an important limitation of our study is not having conducted a secondary 24-h recalls to allow for adjustment for intra-individual variation. Thirdly, it should be of interest that dietary assessment of free sugars was validated by biomarkers of added sugar intake [43]. Unfortunately, data on novel biomarkers of added sugar intake was not available in our study. Despite these limitations, our study also had some strengths. As far as we know, this is the first study to examine the relationship between consumption of free sugars and clinical disease measures in a population of SLE patients. Secondly, our study included a well-characterised cohort of the population with SLE, including patients in an early-stage of the disease and excluding any with lupus severe complications or affected by other autoimmune diseases. Another important strength was the fact that we considered the use of medications (including antimalarials, immunosuppressors, and corticosteroids) as well as age, sex, and energy intake, as cofounders because these factors could have influenced the results.

These findings are of clinical significance because they highlight the important role of sugar consumption in the prognosis of SLE. Thus, effective education strategies for SLE patients should be designed with the aim of minimising the consumption of free sugars by empowering patients to identify and avoid foods and beverages rich in free sugars. This, and the promotion of nutrition programs, represents a valuable preventive strategy that could help improve the prognosis of SLE patients when implemented alongside their usual pharmacological treatments.

## 5. Conclusions

In conclusion, our results demonstrated that patients with active SLE consumed higher amounts of free sugars than those with inactive SLE. We found significant associations between the intake of free sugar and disease activity, the number of disease complications, and the presence of dyslipidaemia. Together this evidence supports the hypothesis that the intake of free dietary sugars might negatively impact SLE disease activity. However, future longitudinal research on SLE patients, including dietary intervention trials, will be required to confirm these preliminary data.

## Figures and Tables

**Table 1:** Descriptive of the main characteristics of the study population classified as, active/inactive systemic lupus erythematosus (SLE) and clinical remission.

Characteristics	Total (*n* = 193)	Active SLE ^a^ (*n* = 32)	Inactive SLE ^a^ (*n* = 161)	Clinical Remission (*n* = 24)	*p* Value (Active vs. Inactive)	*p* Value (Active vs. Remission)
Female	174 (90.2)	29 (90.6)	145 (90.1)	21 (87.5)	0.922	0.708
Age (years)	48.25 ± 12.54	46.09 ± 12.96	48.69 ± 12.45	50.25 ± 12.53	0.287	0.232
BMI (kg/m^2^)	26.74 ± 5.87	27.51 ± 6.19	26.58 ± 5.82	25.65 ± 4.69	0.414	0.205
Clinical Data						
Number of complications	3.67 ± 1.41	3.87 ± 1.49	3.63 ± 1.39	3.08 ± 1.05	0.406	0.027
SLEDAI ^a^ score	2.37 ± 2.64	7.13 ± 1.98	1.41 ± 1.47	0.00 ± 0.00	<0.001	<0.001
SDI score	0.86 ± 1.27	1.46 ± 1.48	0.74 ± 1.20	0.54 ± 0.97	0.003	0.007
hsCRP (mg/dL)	2.89 ± 3.54	2.98 ± 3.31	2.87 ± 3.60	2.68 ± 3.37	<0.001	0.750
Hcy (µmol/L)	12.20 ± 5.80	11.84 ± 4.21	12.28 ± 6.07	12.59 ± 5.02	0.719	0.579
Anti-dsDNA (IU/mL)	20.56 ± 46.40	47.70 ± 77.94	15.34 ± 35.51	0.00 ± 0.00	0.878	0.002
Complement C3 level (mg/dL)	109.62 ± 27.77	110.00 ± 32.50	109.55 ± 26.84	113.92 ± 16.81	0.933	0.561
Complement C4 level (mg/dL)	22.70 ± 10.88	19.88 ± 11.46	23.27 ± 10.70	24.59 ± 6.36	0.108	0.056
Medication Used						
Antimalarial use	142 (74.0)	24 (77.4)	118 (73.3)	18 (75.0)	0.299	0.834
Immunosuppressor use	65 (33.9)	13 (41.9)	52 (32.3)	7 (29.2)	0.632	0.329
Corticoid use	75 (39.1)	16 (51.6)	59 (36.6)	8 (33.3)	0.118	0.175
Cardiovascular Risk Factors						
Hypertension *n* (%)	64 (33.2)	10 (31.3)	54 (33.5)	4 (16.7)	0.802	0.212
Diabetes *n* (%)	3 (1.6)	0 (0)	3 (1.9)	0 (0)	0.436	-
Obesity *n* (%)	114 (59.4)	20 (64.5)	94 (58.4)	15 (62.5)	0.524	0.877
Dyslipidemia n (%)	67 (34.7)	10 (31.3)	57 (35.4)	8 (33.3)	0.652	0.869
TC (mg/dL)	185.07 ± 45.71	189.27 ± 44.43	184.25 ± 46.05	186.33 ± 39.02	0.570	0.795
TG (mg/dL)	103.38 ± 56.17	108.29 ± 48.73	102.42 ± 57.60	89.26 ± 43.74	0.555	0.138
HDL-C (mg/dL)	56.54 ± 14.78	52.40 ± 12.37	57.34 ± 15.11	58.91 ± 12.17	0.056	0.056
LDL-C (mg/dL)	109.43 ± 34.98	113.29 ± 40.31	108.68 ± 33.94	109.25 ± 36.00	0.554	0.696
SBP (mmHg)	121.63 ± 18.70	128.80 ± 14.56	120.75 ± 19.04	122.46 ± 16.89	0.136	0.346
DBP (mmHg)	84.98 ± 19.11	89.00 ± 10.66	84.50 ± 19.88	86.61 ± 6.97	0.485	0.549
ABI	0.99 ± 0.12	0.99 ± 0.14	0.98 ± 0.12	1.00 ± 0.10	0.988	0.590
Nutrients						
Energy (kcal)	1726.02 ± 495.77	1769.46 ± 502.49	1717.39 ± 495.55	1664.58 ± 403.34	0.589	0.390
Total carbohydrates (g)	179.52 ± 61.09	196.84 ± 56.81	176.08 ± 61.49	159.66 ± 45.82	0.079	0.009
Proteins (g)	72.23 ± 26.96	65.87 ± 21.49	73.49 ± 27.80	78.12 ± 27.31	0.145	0.651
Fats (g)	81.00 ± 31.72	81.81 ± 36.62	80.84 ± 30.78	77.92 ± 27.62	0.875	0.138
Starch (g)	95.13 ± 39.86	97.59 ± 46.56	94.64 ± 38.53	87.00 ± 38.60	0.703	0.357
Total sugars (g)	75.37 ± 35.36	85.34 ± 31.62	73.39 ± 35.82	68.70 ± 27.93	0.081	0.042
Intrinsic sugars (g)	45.47 ± 29.35	46.62 ± 23.76	45.25 ± 30.39	36.98 ± 17.36	0.809	0.085
Free sugars (g)	29.89 ± 25.47	38.71 ± 28.87	28.31 ± 24.43	31.72 ± 23.62	0.035	0.324
Free sugars (%)	6.73 ± 5.00	8.60 ± 5.51	6.36 ± 4.82	7.42 ± 5.37	0.020	0.424

Data are expressed as mean and range or frequency and percentage. T-students tests (continuous variables) or chi squared tests (categorical variables). ^a^ Data were distributed in active SLE (SLEDAI ≥ 5), inactive SLE (SLEDAI < 5) and clinical remission (DORIS framework). BMI = body mass index; SLE = systemic lupus eritematosus; SLEDAI = systemic lupus erythematosus disease activity index; SDI = damage index for systemic lupus erythematosus; hsCRP = high-sensitivity C-reactive protein; Hcy = homocysteine; Anti-dsDNA = Anti-double stranded DNA antibodies; TC = total cholesterol; TG = triglycerides; HDL = high density lipoprotein; LDL = low density lipoprotein; SBP = Systolic blood pressure; DBP = diastolic blood pressure; ABI= ankle brachial index.

**Table 2:** Descriptive of the main characteristics the study population classified as, active/inactive SLE and clinical remission according to gender.

Characteristics	Total (*n* = 193)		Active SLE ^a^ (*n* = 32)		Inactive SLE ^a^ (*n* = 161)		Clinical Remission (*n* = 24)		*p* Value (Active vs. Inactive)		*p* Value (Active vs. Remission)	
	Females (*n* = 174)	Males (*n* = 19)	Females (*n* = 29)	Males (*n* = 3)	Females (*n* = 145)	Males (*n* = 16)	Females (*n* = 21)	Males (*n* = 3)	Females (*n* = 174)	Males (*n* = 19)	Females (*n* = 50)	Males (*n* = 6)
Age (years)	47.77 ± 12.56	52.68 ± 11.79	45.55 ± 11.74	51.33 ± 25.02	48.21 ± 12.71	52.94 ± 8.99	49.76 ± 13.20	53.67 ± 6.65	0.299	0.835	0.241	0.884
BMI (kg/m^2^)	26.61 ± 6.04	27.91 ± 3.99	27.32 ± 6.35	29.35 ± 4.85	26.47 ± 5.99	27.64 ± 3.93	25.72 ± 4.72	25.14 ± 5.44	0.487	0.512	0.333	0.374
Clinical Data												
Number of complications	3.68 ± 1.46	3.63 ± 0.89	3.92 ± 1.56	3.33 ± 0.57	3.63 ± 1.44	3.68 ± 0.94	3.00 ± 1.09	3.66 ± 0.57	0.331	0.545	0.024	0.519
SLEDAI^a^ score	2.39 ± 2.63	2.21 ± 2.74	7.07 ± 1.96	7.67 ± 2.51	1.45 ± 1.51	1.19 ± 1.04	0	0	<0.001	<0.001	<0.001	0.006
SDI score	0.83 ± 1.29	1.15 ± 1.11	1.37 ± 1.47	2.33 ± 1.52	0.72 ± 1.22	0.93 ± 0.92	0.47 ± 0.98	1.00 ± 1.00	0.012	0.044	0.018	0.275
hsCRP (mg/dL)	2.66 ± 3.24	4.90 ± 5.28	2.67 ± 3.32	5.80 ± 1.47	2.66 ± 3.24	4.73 ± 5.74	2.43 ± 2.87	4.45 ± 6.55	0.985	0.758	0.791	0.746
Hcy (µmol/L)	11.89 ± 5.68	15.60 ± 6.16	11.29 ± 3.88	18.62 ± 0.65	12.01 ± 5.98	15.09 ± 6.54	12.17 ± 5.09	16.75 ± 0.35	0.569	0.476	0.514	0.071
Anti-dsDNA (IU/mL)	19.91 ± 43.32	26.44 ± 69.81	49.49 ± 80.87	31.00 ± 48.50	14.20 ± 28.49	25.59 ± 74.36	0	0	<0.001	0.906	0.007	0.330
Complement C3 level (mg/dL)	110.82 ± 27.23	98.72 ± 30.91	110.10 ± 32.27	109.06 ± 42.27	110.97 ± 26.23	96.78 ± 29.72	116.81 ± 15.65	93.66 ± 9.86	0.876	0.543	0.383	0.572
Complement C4 level (mg/dL)	22.79 ± 11.08	21.94 ± 9.03	19.25 ± 10.72	25.96 ± 19.11	23.50 ± 11.05	21.18 ± 6.74	25.39 ± 6.37	19.03 ± 2.68	0.059	0.416	0.024	0.568
Medication Used												
Antimalarial use	126 (72.3)	17 (89.5)	22 (78.6)	2 (66.7)	103 (71.0)	15 (93.8)	15 (71.4)	3 (100)	0.415	0.161	0.565	0.273
Immunosuppressor use	58 (33.5)	7 (36.8)	12 (42.9)	1 (33.3)	46 (31.7)	6 (37.5)	7 (33.3)	0 (0)	0.253	0.891	0.498	0.273
Corticoid use	66 (38.2)	9 (47.4)	14 (50.0)	2 (66.7)	52 (35.9)	7 (43.8)	7 (33.3)	1 (33.3)	0.159	0.466	0.243	0.414
Cardiovascular risk factors												
Hypertension n (%)	55 (31.6)	9 (47.4)	9 (31.0)	1 (33.3)	46 (31.7)	8 (50.0)	4 (19.0)	0 (0)	0.942	0.281	0.340	0.273
Diabetes n (%)	1 (0.6)	2 (10.5)	0 (0)	0 (0)	1 (0.7)	2 (12.5)	0 (0)	0 (0)	0.654	0.517	-	-
Obesity n (%)	100 (57.5)	14 (77.8)	18 (62.1)	2 (66.7)	82 (56.6)	12 (75.0)	13 (61.9)	2 (66.7)	0.583	0.423	0.991	0.361
Dyslipidemia n (%)	62 (35.6)	5 (26.3)	9 (31.0)	1 (33.3)	53 (36.6)	4 (25.0)	7 (33.3)	1 (33.3)	0.571	0.764	0.863	1
TC (mg/dL)	185.88 ± 46.69	177.72 ± 35.78	191.09 ± 44.68	172.33 ± 46.71	184.87 ± 47.16	178.73 ± 35.20	186.09 ± 40.38	188.00 ± 34.59	0.521	0.785	0.688	0.665
TG (mg/dL)	101.76 ± 53.91	117.97 ± 73.66	102.79 ± 47.11	159.66 ± 35.64	101.55 ± 55.30	110.16 ± 77.00	92.10 ± 46.29	70.33 ± 8.50	0.912	0.298	0.439	0.013
HDL-C (mg/dL)	56.56 ± 14.43	56.40 ± 18.17	52.83 ± 12.04	48.33 ± 17.61	57.28 ± 14.77	57.91 ± 18.42	58.19 ± 12.31	64.00 ± 12.00	0.136	0.418	0.134	0.272
LDL-C (mg/dL)	110.26 ± 35.09	101.89 ± 33.91	115.54 ± 38.89	92.33 ± 56.72	109.23 ± 34.36	103.68 ± 30.49	109.14 ± 37.53	110.00 ± 28.51	0.386	0.609	0.566	0.655
SBP (mmHg)	120.62 ± 19.09	130.88 ± 11.75	131.25 ± 15.35	119.00 ± 4.24	119.49 ± 19.18	134.28 ± 10.98	121.66 ± 17.39	132.00 ± 0	0.098	0.106	0.223	0.242
DBP (mmHg)	85.04 ± 19.93	84.44 ± 8.94	92.37 ± 8.91	75.50 ± 3.53	84.26 ± 20.65	87.00 ± 8.38	85.91 ± 6.78	95.00 ± 0	0.277	0.111	0.082	0.139
ABI	0.97 ± 0.12	1.08 ± 0.15	0.97 ± 0.12	1.22 ± 0.19	0.98 ± 0.12	1.06 ± 0.14	1.00 ± 0.10	1.03 ± 0.10	0.805	0.191	0.384	0.247
Nutrients												
Energy (kcal)	1672.73 ± 472.71	2214.05 ± 441.79	1760.48 ± 521.77	1865.33 ± 299.84	1655.18 ± 462.24	2281.12 ± 437.99	1625.19 ± 409.62	1940.33 ± 250.16	0.275	0.130	0.328	0.728
Total carbohydrates (g)	174.20 ± 59.07	228.35 ± 58.96	197.72 ± 58.93	188.33 ± 35.79	169.49 ± 58.17	235.81 ± 60.17	155.66 ± 41.12	215.66 ± 43.14	0.018	0.209	0.003	0.446
Proteins (g)	71.17 ± 26.50	91.19 ± 24.24	65.34 ± 22.04	71.00 ± 17.57	71.13 ± 27.27	94.87 ± 23.83	77.09 ± 29.45	89.33 ± 0.57	0.284	0.120	0.113	0.145
Fats (g)	78.43 ± 30.92	104.52 ± 30.00	80.58 ± 38.08	93.66 ± 15.82	78.00 ± 29.42	106.56 ± 31.91	77.61 ± 29.32	85.33 ± 18.61	0.683	0.510	0.767	0.586
Starch (g)	91.83 ± 39.49	125.36 ± 29.72	97.06 ± 48.76	102.66 ± 16.19	90.78 ± 37.48	129.62 ± 30.05	84.19 ± 40.19	106.66 ± 17.89	0.436	0.155	0.327	0.788
Total sugars (g)	74.23 ± 35.61	85.78 ± 32.00	87.96 ± 30.74	60.00 ± 34.59	71.48 ± 35.98	90.62 ± 30.19	63.38 ± 24.03	106.00 ± 28.58	0.023	0.132	0.004	0.150
Intrisinc sugars (g)	45.18 ± 30.05	48.16 ± 22.30	47.18 ± 23.68	41.20 ± 29.16	44.78 ± 31.23	49.47 ± 21.72	35.74 ± 17.54	45.67 ± 16.07	0.695	0.571	0.067	0.828
Free sugars (g)	74.23 ± 35.61	37.62 ± 22.76	40.77 ± 29.45	18.79 ± 11.14	26.89 ± 24.26	41.15 ± 22.83	27.63 ± 21.94	60.33 ± 14.25	0.007	0.121	0.091	0.016
Free sugars (%)	6.72 ± 5.09	6.83 ± 4.19	9.09 ± 5.54	3.85 ± 2.02	6.24 ± 4.88	7.38 ± 4.29	6.71 ± 5.34	12.39 ± 1.96	0.006	0.188	0.134	0.006

Data are expressed as mean and range or frequency and percentage. T-students tests (continuous variables) or chi squared tests (categorical variables). ^a^ Data were distributed in active SLE (SLEDAI ≥ 5), inactive SLE (SLEDAI < 5) and clinical remission (DORIS framework). BMI = body mass index; SLE = systemic lupus eritematosus; SLEDAI = systemic lupus erythematosus disease activity index; SDI = damage index for systemic lupus erythematosus; hsCRP = high-sensitivity C-reactive protein; Hcy = homocysteine; Anti-dsDNA = Anti-double stranded DNA antibodies; TC = total cholesterol; TG = triglycerides; HDL= high density lipoprotein; LDL = low density lipoprotein; SBP = Systolic blood pressure; DBP= diastolic blood pressure; ABI = ankle brachial index.

**Table 3:** Beta estimates and confidence intervals for the association between free sugars intake and clinical disease activity parameters in SLE patients.

	Free Sugars (g)						Free Sugars (%)					
	Overall Population		Females (*n* = 174)		Males (*n* = 19)		Overall Population		Females (*n* = 174)		Males (*n* = 19)	
Clinical Parameters	β (95% CI)	*p* Value	β (95% CI)	*p* Value	β (95% CI)	*p* Value	β (95% CI)	*p* Value	β (95% CI)	*p* Value	β (95% CI)	*p* Value
**Number of Complications**	0.009 (0.001, 0.018)	0.033	0.010 (0.000, 0.019)	0.039	0.004 (−0.021, 0.029)	0.752	0.046 (0.008, 0.084)	0.018	0.048 (0.007, 0.089)	0.021	0.016 (−0.120, 0.153)	0.798
**SLEDAI Score**	0.017 (0.001, 0.034)	0.043	0.023 (0.005, 0.041)	0.012	−0.036 (−0.085, 0.013)	0.132	0.086 (0.011, 0.161)	0.024	0.109 (0.032, 0.187)	0.006	−0.207 (0.467, 0.054)	0.109
**SDI Score**	0.002 (−0.006, 0.009)	0.687	0.002 (−0.007, 0.010)	0.713	−0.004 (−0.030, 0.021)	0.723	0.006 (−0.029, 0.041)	0.729	0.007 (−0.029, 0.043)	0.702	−0.024 (−0.163, 0.115)	0.711
**hsCRP (mg/dL)**	−0.001 (−0.024, 0.021)	0.901	−0.012 (−0.034, 0.009)	0.264	0.084 (−0.057, 0.225)	0.217	−0.039 (−0.138, 0.061)	0.445	−0.076 (−0.172, 0.019)	0.114	0.434 (−0.337, 1.206)	0.244
**Hcy (µmol/L)**	−0.005 (−0.045, 0.034)	0.785	−0.004 (−0.045, 0.036)	0.834	0.044 (−0.183, 0.270)	0.663	−0.023 (−0.196, 0.150)	0.795	−0.018 (−0.195, 0.159)	0.841	0.244 (−0.986, 1.474)	0.653
**Anti-dsDNA (IU/mL)**	0.073 (−0.229, 0.374)	0.636	0.148 (−0.150, 0.447)	0.327	−0.702 (−2.608, 1.203)	0.438	0.321 (−1.025, 1.667)	0.638	0.633 (−0.671, 1.936)	0.339	−3.845 (−14.197, 6.507)	0.434
**Complement C3 Level (mg/dL)**	−0.066 (−0.244, 0.112)	0.464	−0.069 (−0.255, 0.117)	0.466	−0.250 (−0.936, 0.436)	0.442	−0.150 (−0.946, 0.646)	0.710	−0.137 (−0.952, 0.678)	0.741	−1.465 (−5.176, 2.245)	0.406
**Complement C4 Level (mg/dL)**	−0.059 (−0.129, 0.012)	0.103	−0.069 (−0.145, 0.008)	0.078	0.025 (−0.225, 0.275)	0.832	−0.312 (−0.625, 0.002)	0.052	−0.342 (−0.675, −0.010)	0.044	0.063 (−1.300, 1.426)	0.922

SLE = systemic lupus eritematosus; SLEDAI = systemic lupus erythematosus disease activity index; SDI = damage index for systemic lupus erythematosus; hsCRP = high-sensitivity C-reactive protein; Hcy = homocysteine; Anti-dsDNA = Anti-double stranded DNA antibodies. Data were adjusted by age, sex, energy intake and medical treatment (immunosuppressor, corticoid and antimalarial use).

**Table 4:** Beta estimates and confidence intervals for the association between free sugars intake and cardiovascular risk factors in SLE patients.

	Free Sugars (g)						Free Sugars (%)					
	Overall Population		Females (*n* = 174)		Males (*n* = 19)		Overall Population		Females (*n* = 174)		Males (*n* = 19)	
Clinical Parameters	β (95% CI)	*p* Value	β (95% CI)	*p* Value	β (95% CI)	*p* Value	β (95% CI)	*p* Value	β (95% CI)	*p* Value	β (95% CI)	*p* Value
**Hypertension**	0.000 (−0.002, 0.003)	0.812	0.000 (−0.003, 0.004)	0.757	0.000 (−0.013, 0.014)	0.960	−0.001 (−0.014, 0.012)	0.872	−0.002 (−0.015, 0.012)	0.828	0.000 (−0.073, 0.073)	0.999
**Diabetes**	0.000 (0.000, 0.001)	0.542	0.000 (−0.001, 0.001)	0.938	0.002 (−0.007, 0.011)	0.661	0.001 (−0.003, 0.004)	0.656	0.000 (−0.002, 0.002)	0.905	0.011 (−0.037, 0.060)	0.623
**Obesity**	−0.460 (−0.228, 7.308)	0.907	−0.001 (−0.005, 0.002)	0.487	−0.002 (−0.021, 0.008)	0.682	−0.527 (−2.062, 1.008)	0.499	−0.007 (−0.021, 0.008)	0.380	−0.012 (−0.066, 0.041)	0.620
**Dyslipidemia**	−0.003 (−0.005, 0.000)	0.024	−0.004 (−0.007, 0.000)	0.026	0.002 (−0.008, 0.013)	0.682	−0.015 (−0.028, −0.002)	0.021	−0.015 (−0.028, −0.001)	0.033	0.009 (−0.048, 0.066)	0.737
**TC (mg/dL)**	−0.155 (−0.420, 0.110)	0.250	−0.154 (−0.483, 0.175)	0.357	−0.382 (−1.172, 0.407)	0.312	−0.623 (−1.971, 0.708)	0.353	−0.607 (−2.045, 0.832)	0.406	−1.665 (−6.026, 2.695)	0.422
**TG (mg/dL)**	0.082 (−0.239, 0.403)	0.615	0.185 (−0.189, 0.559)	0.331	0.236 (−1.633, 2.106)	0.788	0.572 (−1.066, 2.209)	0.492	0.581 (−1.067, 2.230)	0.487	0.158 (−10.035, 13.350)	0.974
**HDL-C (mg/dL)**	−0.057 (−0.142, 0.029)	0.192	−0.072 (−0.174, 0.030)	0.164	0.173 (−0.272, 0.617)	0.414	−0.152 (−0.584, 0.281)	0.490	−0.224 (−0.670, 0.222)	0.323	1.085 (−1.308, 3.478)	0.343
**LDL-C (mg/dL)**	−0.181 (−0.384, 0.022)	0.080	−0.169 (−0.417, 0.079)	0.181	−0.443 (−1.206, 0.321)	0.231	−0.866 (−1.893, 0.161)	0.098	−0.762 (−1.846, 0.321)	0.167	−1.904 (−6.156, 2.348)	0.349
**SBP (mmHg)**	−0.057 (−0.225, 0.110)	0.498	−0.063 (−0.267, 0.141)	0.542	0.034 (−0.943, 1.010)	0.920	−0.201 (−0.965, 0.563)	0.602	−0.206 (−1.046, 0.635)	0.627	0.151 (−4.846, 5.148)	0.929
**DBP (mmHg)**	−0.021 (−0.209, 0.168)	0.828	−0.035 (−0.269, 0.199)	0.767	0.055 (−0.806, 0.915)	0.853	−0.016 (−0.878, 0.845)	0.970	−0.071 (−1.034, 0.891)	0.883	0.389 (−3.986, 4.764)	0.796
**ABI**	0.000 (−0.001, 0.001)	0.694	0.000 (−0.001, 0.001)	0.534	−0.002 (−0.006, 0.002)	0.259	0.000 (−0.004, 0.004)	0.845	0.000 (−0.004, 0.004)	0.905	−0.011 (−0.033, 0.011)	0.293

SLE = systemic lupus eritematosus; TC = total cholesterol; TG = triglycerides; HDL = high density lipoprotein; LDL = low density lipoprotein; SBP = Systolic blood pressure; DBP = diastolic blood pressure; ABI = ankle brachial index. Data were adjusted by age, sex, energy intake and medical treatment (immunosuppressor, corticoid and antimalarial use).
